# Senescent-like Blood Lymphocytes and Disease Progression in Amyotrophic Lateral Sclerosis

**DOI:** 10.1212/NXI.0000000000200042

**Published:** 2022-11-02

**Authors:** Ozlem Yildiz, Johannes Schroth, Timothy Tree, Martin R. Turner, Pamela J. Shaw, Sian M. Henson, Andrea Malaspina

**Affiliations:** From the Neuroscience and Trauma Centre (O.Y., A.M.), Blizard Institute, Barts and The London School of Medicine & Dentistry, Queen Mary University of London; Queen Square Motor Neuron Disease Centre (A.M.), Neuromuscular Department, Institute of Neurology, University College London; Translational Medicine and Therapeutics (J.S., S.M.H.), William Harvey Research Institute, Barts and the London, Queen Mary University of London; Department of Immunobiology (T.T.), School of Immunology & Microbial Sciences, King's College London; Nuffield Department of Clinical Neurosciences (M.R.T.), University of Oxford; and Sheffield Institute for Translational Neuroscience (P.J.S.), University of Sheffield, UK.

## Abstract

**Background and Objectives:**

Aging is known to exacerbate neuroinflammation, and in the neurodegenerative disorder amyotrophic lateral sclerosis (ALS), an older age is associated with a worse prognosis. We have previously shown the activation of cell senescence pathways in the proteome of peripheral blood mononuclear cells and the increase of proinflammatory cytokines in blood from individuals living with ALS. In this single-center, retrospective study, we investigated the expression of senescent-like blood mononuclear cells in ALS.

**Methods:**

We first applied multidimensional cytometry by time-of-flight (CyTOF) to study the senescent immunophenotype of blood mononuclear cells from 21 patients with ALS and 10 healthy controls (HCs). We then used targeted flow cytometry (FC) to investigate frequencies of senescent blood lymphocytes in 40 patients with ALS and 20 HCs. Longitudinal analysis included 2 additional time points in 17 patients with ALS. Frequencies of senescent-like lymphocytes were analyzed in relation to survival.

**Results:**

Unsupervised clustering of CyTOF data showed higher frequencies of senescent CD4^+^CD27^−^CD57^+^ T cells in patients with ALS compared with those in HCs (*p* = 0.0017, false discovery (FDR)-adjusted *p* = 0.029). Moderate to strong negative correlations were identified between CD4 T central memory–cell frequencies and survival (R = −061, *p* = 0.01; FDR-adjusted *p* < 0.1) and between CD95 CD8 cells and ALS functional rating scale revised at baseline (R = −0.72, *p* = 0.001; FDR-adjusted *p* < 0.1).Targeted FC analysis showed higher memory T regulatory cells (*p* = 0.0052) and memory CD8^+^ T cell (M-Tc; *p* = 0.0006) in bulbar ALS (A-B) compared with those in limb ALS (A-L), while late memory B cells (LM-B) were also elevated in A-B and fast-progressing ALS (*p* = 0.0059). Higher M-Tc levels separated A-B from A-L (AUC: 0.887; *p* < 0.0001). A linear regression model with prespecified clinical independent variables and neurofilament light chain plasma concentration showed that higher frequencies of LM-B predicted a shorter survival (hazard ratio: 1.094, CI: 1.026–1.167; *p* = 0.006).

**Discussion:**

Our data suggest that a systemic elevation of senescent and late memory T and B lymphocytes is a feature of faster progressing ALS and of ALS individuals with bulbar involvement. Lymphocyte senescence and their memory state may be central to the immune dysregulation known to drive disease progression in ALS and a target for biomarkers and therapeutics discovery.

Amyotrophic lateral sclerosis (ALS) is the most prevalent motor neuron disease, with a lifetime risk of 1 in 300 in the UK.^1^ An example of the clinical heterogeneity of ALS is the progression to end-stage disease from onset of symptoms that could be anything from a few months to more than a decade.^2^ Uncertainties surrounding prognosis and the pathobiology underpinning the onset and propagation of ALS are the main obstacles for the development of effective therapeutics.^1^ The integration of biomarkers of prognosis and disease progression is therefore the key requirement to move away from expensive and large cohort size clinical trials.

Multiple clinical and genetic factors have been identified as prognostic indicators of ALS, including age at onset, clinical features of frontotemporal and bulbar functions impairment, body mass index, male predominance, the diagnostic delay, the A4V mutation of the superoxide dismutase 1 (*SOD1*) gene, the C9orf72 gene repeat expansion, and variations in the ATX2 and UNC13A genes.^1,3-5^ In addition, changes in the immune response such as the reduction of CD4^+^FOXP3^+^ T regulatory cells (Tregs) is associated with a faster progressing disease.^6-8^ Our unbiased proteomic analysis of plasma and peripheral blood mononuclear cells (PBMCs) in blood samples from patients with ALS has shown the activation of molecular pathways involved in immunoregulation and cell senescence in faster progressing ALS (A-F) and at a later stage of disease.^9^ We and others have also reported an increased blood and CSF concentration of proinflammatory cytokines in individuals with ALS.^10-13^ These inflammatory mediators are enumerated within the senescence-associated secretory phenotype (SASP) and have been described in age-related immune dysregulation.^14^ The SASP inflammatory microenvironment spreads the tissue-disrupting effect of senescence regionally and systemically, impairing the function of other immune cells. Critically, other alterations observed in aging and senescence are aligned with brain-specific changes seen in ALS, including a disturbed autophagy/lysosomal protein degradation, altered RNA splicing, and errors in nuclear-cytoplasmic transport.^15-18^ We can therefore hypothesize a potential role for cell senescence in the immunologic dysregulation identified in ALS.

Immunosenescence is a multifaceted process where an impaired immune response to new antigens coupled with a greater propensity for autoimmunity leads to a constitutive low-grade inflammation. In T cells, cell senescence manifests with the loss of CD27 and CD28 together with the acquisition of CD57, of markers such as the Killer Cell Lectin-Like Receptor G1 (KLRG1), with impaired virus control and reduced cross-protection to immunologic challenges.^19^ In addition, immunosenescence leads to changes of B-cell responses, with a decreased differentiation and output of mature B cells and the rise of highly inflammatory late memory B cells (LM-B), SASP-producing cells that hamper the ability of other B cells to proliferate and generate optimal antibody responses.^21^ B-cell senescence results in a reduced generation of high-affinity, protective antibodies.^20^

In this study, we used a 2-stage approach to first investigate the immunophenotype of PBMCs from individuals living with ALS and then focused on lymphocytes expressing known features of immunosenescence. We showed that lymphocytes from patients with ALS are skewed toward a senescent and late memory state when compared with those from age-matched healthy controls (HCs).

## Methods

### Clinical and Demographic Characteristics

Sixty-one patients with ALS with a diagnosis of ALS based on standard diagnostic criteria^22^ were consented for inclusion in the ALS Biomarker study (09/H0703/27) and AMBRoSIA (16/LO/2136). Blood samples were taken by venipuncture from all patients with ALS at baseline (visit 1: V1), at or shortly after diagnosis, and at 2 consecutive time points (visit 2: V2 and visit 3: V3) in 17 patients with ALS. The median time interval between V1 and V2 was 6.54 months (interquartile range [IQR] 4.83–8.28) and 7.21 months (IQR 5.31–9.26) from V2 to V3. Samples were taken at a single time point from 30 age-matched and sex-matched HCs. Exclusion criteria for participants were a history of autoimmune diseases, recent trauma, and chronic and systemic inflammatory disorders. The ALS functional rating scale revised (ALSFRS-R) was used to rate neurologic disability.^23^

### Separation and Preparation of Human PBMCs

The extraction from blood, processing, and storage of PBMCs is reported in eMethods, links.lww.com/NXI/A750. Cryopreserved PBMCs were thawed at room temperature and resuspended in 10 mL of RPMI 1640 medium (Thermo Fisher Scientific) at 37°C, supplemented with 10% FBS and 1% penicillin streptomycin (VWR International Ltd). For cytometry by time-of-flight (CyTOF), cells were lysed using Pierce Universal nuclease (Thermo Fisher Scientific) and diluted 1:10.000 (25 U/mL final concentration). For flow cytometry (FC) and CyTOF staining, the cell suspension was twice centrifuged at 483*g* for 10 minutes at room temperature and the pellet resuspended in Roswell Park Memorial Institute Medium 1640 medium.

Cryopreservation time (in days), defined as the interval between the time of PBMC extraction, liquid nitrogen immersion, and the time of thawing for use in cytometry experiments (in days), was noted for each experiment and used to identify any loss of viable cells with longer storage periods. To investigate the effect of cryostorage on cell viability, PBMCs (30 million cells) were extracted from 3 HCs and 4 patients with ALS (mean age: 64.29 years; SD: 6.40; 4 males and 3 females) immediately after phlebotomy. Fifteen million PBMCs were used to investigate by FC the relative percentage of live (CD45) cells, total lymphocytes, CD3 cells, and CD19 cells. The remaining 15 million PBMCs were cryostored and later thawed to undertake the same FC analysis (mean storage time: 67.29; SD 78.45).

### Staining for CyTOF and FC Experiments

Detailed protocols for cell staining used in cytometry experiments are reported in the supplementary data, including the lists of unconjugated primary antibodies used for the FC experiments involving T-cell, B-cell, and senescent-cell panels. Mass cytometry antibody panel and reagents are reported in eTable 3, links.lww.com/NXI/A750. Cells were acquired using the NovoCyte 13-colour flow cytometer configured with 405, 488, and 640 nm lasers for data acquisition and analysis. eTable1, links.lww.com/NXI/A750 reports the markers used to identify T-cell and B-cell subsets and markers expressed in senescent T cells.

### CyTOF Multidimensional Clustering

Flow cytometry files underwent standard preprocessing. After initial quality control, data were normalized, debris and doublets were removed, and gating was performed to obtain live CD45^+^ cells (eFigure 6, links.lww.com/NXI/A750). Manual gating of cells was conducted using FlowJo v10.7.1 to identify the immune cell subsets under investigation (eFigure 1, links.lww.com/NXI/A750).

Unsupervised computational analysis of preprocessed data was performed using R.^24^ In brief, flow cytometry files were *arcsinh* (inverse hyperbolic sine) transformed with a cofactor of 5. Markers were then separated into “lineage-defining markers” (CD16, FOXP3, NCAM, CD38, CD8, CD45RA, interleukin (IL) 3R, CD27, CXCR3, CD28, CD19, Vδ2, CD4, IgD, CD14, CD11c, CD3, CCR7, IL2R, CD57, HLA-DR, and IL7Rα) and “function-defining markers” (CD24, CCR2, CD169, CD61, PD1, CD86, CCR6, CCR4, Fas, CD45, and CD40). Unsupervised clustering was performed using the phenograph algorithm from the FastPG package.^25^ Phenograph was used instead of FowSOM for its superior ability to detect refined subclusters.^26^ Clusters with a frequency of <1% of the analyzed cells were excluded from subsequent analyses. Dimensionality reduction through fast Fourier transform–accelerated interpolation-based t-Stochastic Neighborhood Embedding (FIt-SNE) was performed on 100,000 cells randomly down-sampled to 50,000 per group (HCs and patients with ALS) using the FIt-SNE package.^27^ The hyperparameters were set as follows: perplexity = 1,000; learning rate = 8,333; theta = 0.5; and maximum iterations = 1,000. These were calculated using established methods for optimal hyperparameter selection.^28^ Owing to its robust performance,^29^ differential abundance was analyzed using quasi-likelihood (QL) negative binomial generalized linear models (NB GLM), implemented in the edgeR package.^30^ An NB GLM was fitted, and a QL F-test with a specified contrast was performed to calculate *p* values for each cell type. A batch variable was included as a covariate to account for batch effects. *p* values of unsupervised phenograph clustered cells were FDR adjusted using the Benjamin and Hochberg approach^31^ (total tests performed = 17). The median marker expression of manually gated cells was analyzed using linear mixed-effects models, with batches as a random effect to account for batch effects and *p* values calculated by the χ^2^ test.

### Measurement of Plasma Neurofilament Light Chain

Analysis of neurofilament light chain (NfL) protein expression was undertaken by single-molecule array (Simoa) using a digital immunoassay HD-1 Analyzer (Quanterix; Lexington, MA), as previously reported.^32^

### Statistical Analysis

Nonparametric group analysis was performed using the Kruskal-Wallis test after log transformation of raw values. Corrections for multiple comparisons were performed using Dunn tests controlling for false discovery rate. Survival was calculated as the time from baseline (V1) to permanent assisted ventilation (≥22 hours per day of noninvasive ventilation), tracheostomy, or death. To obtain a measure of the rate of disease progression spanning the whole disease duration, we subtracted the reported ALSFRS-R at baseline from 48 (approximation of healthy state at onset) and divided it by the interval time in months (disease progression rate at baseline: ΔFRS, points/month). The postbaseline rate was obtained by subtracting the last recorded ALSFRS-R score from the baseline ALSFRS-R divided by the time interval (ALSFRS-R change, points/mo). As previously reported, to separate off ALS patient subsets based on disease progression and undertake group analysis of lymphocyte subset expression, ΔFRS and ALSFRS-R change smaller than −1.0 points/mo was used to identify faster progressing ALS (A-F), while greater than −0.5 points/mo was used to identify slower progressing ALS. In bulbar ALS (A-B), bulbar features were the only clinical and neurophysiologic manifestations at onset and at diagnosis, while for limb ALS (A-L), upper and lower motor neuron signs involving the limbs were the only findings at onset and at diagnosis.

A linear model was fitted with predefined variables previously reported as predictors of the rate of disease progression and survival in ALS.^32^ Explanatory variables included sex, age at baseline (V1), A-B, baseline ALSFRS-R, ΔFRS, diagnostic delay, Nf-L, and lymphocyte subset frequencies, while ALSFRS change was the dependent variable. The prognostic value of predefined variables and of lymphocyte subsets frequencies were also evaluated using Cox regression and by univariate Kaplan-Meier analysis with permanent assisted ventilation and tracheostomy-free survival from baseline as outcome.

Analysis of variance (ANOVA) for repeated measures fitting a mixed model with missing values at random was applied for longitudinal analysis and receiver operating characteristic (ROC) curve nonparametric analysis to ascertain whether lymphocyte subset frequencies discriminated between ALS phenotypic variants and between patients with ALS and HCs.

For the CyTOF data, a partial correlation analysis with correction for batch effects was undertaken to look at associations between cell subset frequencies and median marker intensities (MMIs) with disease progression parameters, including ALSFRS at V1, ΔFRS, and survival from onset. Data were analyzed using Prism (Version 8.0, GraphPad software; San Diego, CA) and SPSS.

### Research Ethics and Informed Consent

Ethical approval for the study was obtained for the ALS Biomarkers Study (East London Research Ethics Committee, London, UK–REC reference 09/H0703/27) and for A Multicentre Biomarker Resource Strategy In ALS (“AMBRoSIA,” South-East Research Ethics Committee–16/LO/2136), and informed consent was available from all participants.

### Data Availability

The unpublished raw data used for the analyses detailed in the study are available from the corresponding author on request.

## Results

### Demographic and Clinical Characteristics

For CyTOF analysis, 21 patients with ALS (mean age 63.0 (SD ± 12.8) years, 40% female) and 10 HCs (mean age 54.6 [SD ± 13.9] years, 30% female) were included. The lymphocyte study by FC was undertaken on 40 patients with ALS (mean age 63.9 years [SD ± 11.4]) and 20 age-matched and sex-matched HCs (mean age 60.4 years [±SD 7.1], 50% female) (Table 1). Patients with ALS had a bulbar form of the disease (A-B) in 45% of cases. A-B were age matched to patients with ALS with a spinal form of the disease ALS (A-L) (mean age 63.3 [±SD 11.5] and 64.5 years [±SD 11.6], respectively). All individuals were sampled at inclusion (baseline: V1), while 17 (42.5%) patients with ALS were sampled at a second visit (V2), of which 12 (70.1%) were sampled at a third visit (V3). The median of the time interval from disease onset to V1 was 16.5 months (IQR 14.6), 6.9 (IQR 10.9) months from V1 to V2, and 7.7 months (IQR 14.4) from V2 to V3. The mean ALSFRS-R score at V1 was 35 (±SD 9.60) for all patients with ALS, 32.23 (±SD 10.52) at V2, and 30.67 (±SD 9.50) at V3 for those patients studied longitudinally (Table 1).

**Table 1:**
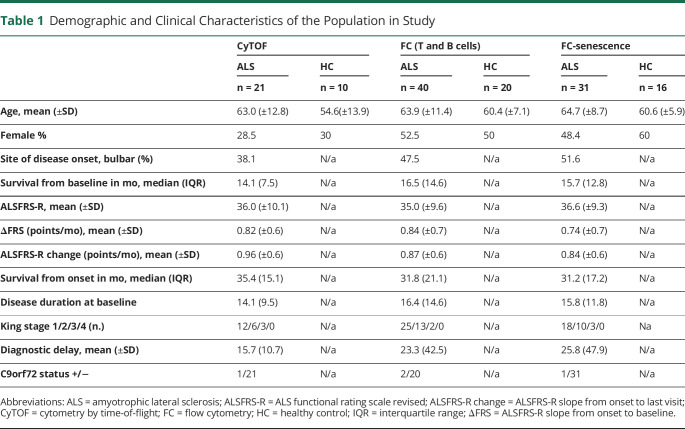
Demographic and Clinical Characteristics of the Population in Study

In the selected patients with ALS, ΔFRS and ALSFRS changes were similar (mean 0.861; SD 0.687 and mean 0.854; SD 0.619, respectively) and strongly correlated (R: 0.952; *p* < 0.0001). To investigate the effect of predefined explanatory variables, ALSFRS change was used as a dependent variable in a linear regression model and ΔFRS was incorporated as an independent variable in the same linear regression and in a Cox prognostic model (Table 2). Analysis of T-cell senescence markers by FC was undertaken on a subset of patients with ALS (n = 31) (mean age 64.7 years [SD ± 8.7], 40% female) and HCs (n = 16) (mean age 60.6 years [SD ± 5.9], 35% female).

**Table 2:**
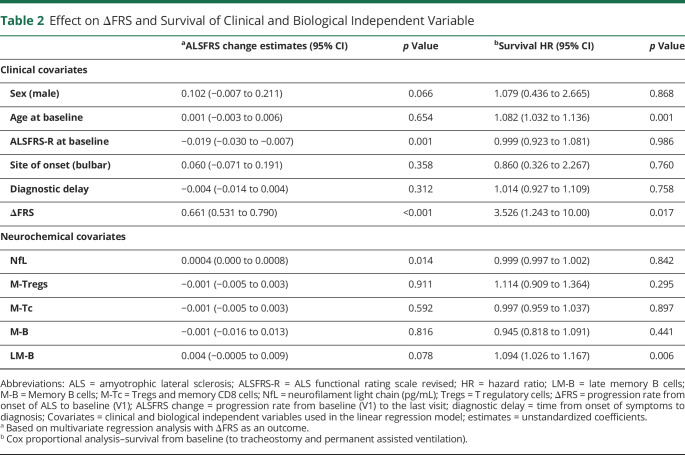
Effect on ΔFRS and Survival of Clinical and Biological Independent Variable

### Effect of Cryopreservation on Cell Viability

We could not find any (negative) correlation between cryopreservation times and the frequencies of B-cell and T-cell subset determined by FC and the MMI of blood mononuclear cells measured by CyTOF (eTable 4, links.lww.com/NXI/A750). These findings suggest that cryostorage time did not affect the viability and the frequency of the mononuclear cells. There was no statistically significant difference between percentages of live cells, total lymphocytes, CD3 cells, and CD19 cells measured by FC in freshly extracted PBMCs and the same cell types measured after variable cryostorage times (data not shown).

### CyTOF Data Analysis: Lymphocyte Profiling by Unsupervised Cell Clustering

**Figure 1:**
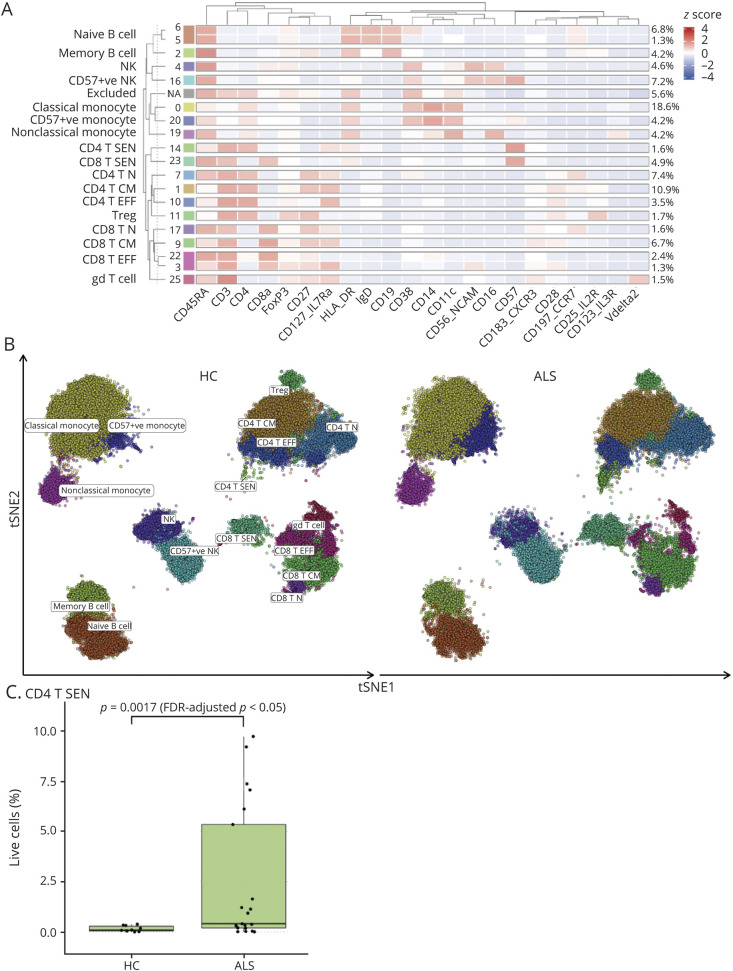
Unsupervised Clustering Analysis Reveals a Senescent Population of CD4 T Cells in Patients With ALS (A) Heatmap showing phenograph clustering of 22 lineage markers identifying 19 clusters with a frequency above the 1% threshold, which were grouped into 17 meta-clusters according to their lineage marker expression. Clusters with a frequency of less than 1% were excluded and grouped into the “Excluded” cluster (NA, 6th row from the top). Cluster frequencies are shown together with their assigned cell type. (B) t-distributed stochastic neighbor embedding (tSNE) of 100,000 down sampled cells for patients with ALS and HCs (50,000 per HC and ALS group), colored by phenograph-determined cell types. (C) Frequency of CD4 senescent T cells (CD4 T SEN) as a percentage of live cells identified by unsupervised phenograph clustering in the HC and ALS groups. *p* values determined through quasi-likelihood negative binomial generalized linear models. ALS = amyotrophic lateral sclerosis; CD4 T SEN = CD4 cells expressing CD57; HC = healthy control.

CyTOF data were analyzed using unsupervised clustering and manual gating independently. The manual gating strategy is detailed in eFigure 2, links.lww.com/NXI/A750.^33^ Unsupervised clustering was conducted using the phenograph algorithm. Twenty-two lineage-defining markers^26^ identified a total of 38 clusters, of which 19 were below a threshold frequency of 1% and were therefore excluded from downstream analysis (Figure 1A). The remaining 19 clusters with a frequency greater than 1% were classified into 17 meta-clusters according to their marker expression (eTable2, links.lww.com/NXI/A750). The frequency of cell populations identified by unsupervised clustering (% live cells) was comparable with those identified using manual gating (eFigure 2, links.lww.com/NXI/A750). We then implemented dimensionality reduction through t-SNE for visualization of cell clusters colored by phenograph-defined meta-clusters. t-SNE of 100,000 sampled cells including 50,000 from HC and ALS samples were colored to define marker-specific cell expression (Figure 1B).

Unsupervised clustering followed by generalized linear modeling, QL F-test, and FDR adjustment revealed a significant difference between patients with ALS and HCs in the abundance of senescent CD4 cells expressing CD57 (CD4 T SEN) (log FC = 3.04, F = 10.01, *p* = 0.0017, FDR-adjusted *p* = 0.029; Figure 1C). The upregulation of CD4 T SEN in patients with ALS compared with that in HCs was also seen using manual gating of samples (data not shown). With manual gating only (eFigure 4, links.lww.com/NXI/A750), naive B cells were less abundant in patients with ALS compared with those in HCs (*p* < 0.005), A-L had more CD4 T-effector (CD4 T EFF) cells than A-B (*p* < 0.05) while CCR4-expressing CD4 T EFF cells were more abundant in blood from A-B compared with those from A-L (*p* < 0.05; eFigure 4, links.lww.com/NXI/A750). Myeloid cells (CD14, CD16, and CD86 nobocytes) were not clustered or differentially regulated, except for a reduction of CD57^+^ CD86 monocytes in patients with ALS compared with that in HCs in manual gating only (*p* < 0.05; eFigure 4, links.lww.com/NXI/A750).

**Figure 2:**
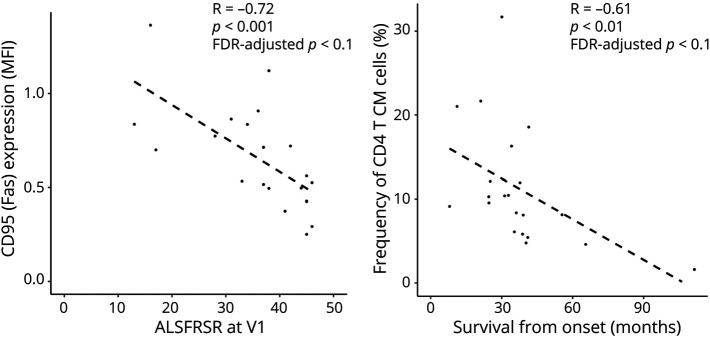
CyTOF Analysis: Association Between Blood Mononuclear Cell Frequencies and Clinical Parameters FDR-adjusted partial correlation analysis correcting for batch effect showed moderate to strong negative correlations between CD4 central memory (CM) cell frequencies and survival and between CD95 (Fas) CD8 cells and ALSFRSR at baseline. ALSFRS-R = amyotrophic lateral sclerosis functional rating scale revised; CyTOF = cytometry by time-of-flight.

FDR-adjusted partial correlation analysis correcting for batch effect showed moderate to strong negative correlations between CD4 central memory (CM; CD3^+^ CD4^+^ CD27^+^ CD45RA^−^) cell frequencies and survival (R = −061, *p* = 0.01; FDR-adjusted *p* < 0.1) and between CD95 (Fas) CD8 cells and ALSFRS-R at baseline (R = −0,72, *p* = 0.001; FDR-adjusted *p* < 0.1) (Figure 2).

A subgroup of patients with ALS had higher levels of CD4 T SEN (Figure 1C). We separated patients with ALS into high and low cell frequencies of CD4 T SEN, using the mean of the overall CD4 T SEN frequency (2.47%). We found no statistically significant difference for age at V1, ALSFRS at V1, ΔFRS, diagnostic delay, sex, and survival from onset between high and low CD4 T SEN cell frequency ALS subgroups.

### FC Analysis

#### Frequencies of T-Cell Subsets in ALS

**Figure 3:**
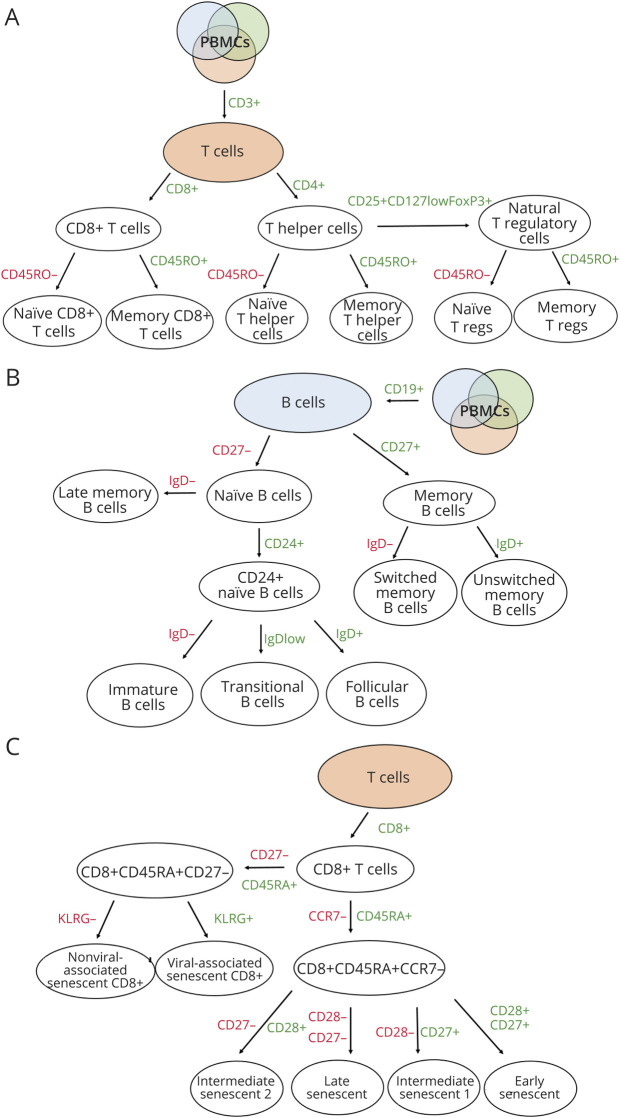
FC Gating Strategy Schematic representation of gating strategy for T cells (A), B cells (B) and T-cell senescence panels (shown for CD8^+^ T cells) (C) used in the FC experiments. FC = flow cytometry.

**Figure 4:**
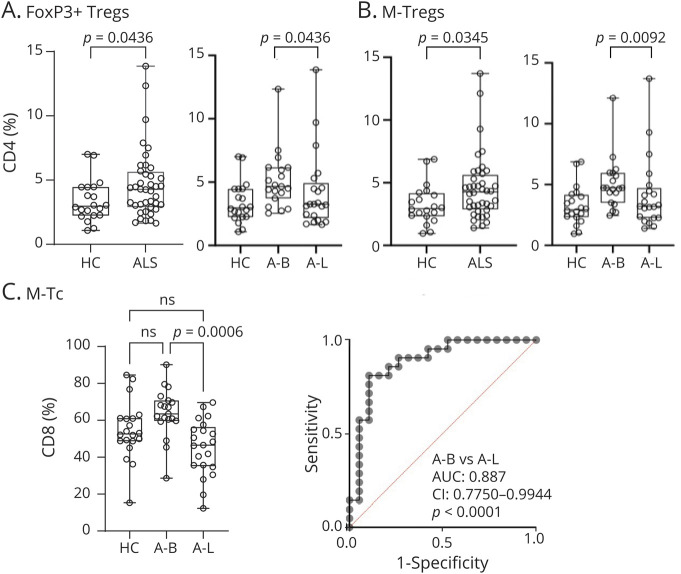
Analysis of T-Cell Subset Frequencies and Association With Site of Onset and Disease Progression in Individuals With ALS (A) FoxP3^+^ Tregs expression is significantly higher in blood from patients with ALS compared with that in HCs. (B) M-Tregs are elevated in ALS and A-B. (C) M-Tc frequencies are significantly upregulated in A-B compared with that in A-L. (D) ROC analysis testing M-Tc ability to separate A-B from A-L. A-B = bulbar ALS; A-L = limb-onset ALS; ALS = amyotrophic lateral sclerosis; HCs = healthy controls; M-Tc = memory CD8^+^ T cells; M-Tregs = memory T regulatory cells; ROC = receiver operating characteristic.

Figure 3 depicts the gating strategy for T and B cells and for T cells expressing senescence markers used for FC data analysis. Frequencies of FoxP3^+^ Tregs and memory Tregs were significantly elevated as a percentage of CD4^+^ cells in patients with ALS compared with those in HCs (*p* = 0.0436 and *p* = 0.0345 respectively; Figure 4, A and B). When patients with ALS were subdivided based on the anatomic site of involvement, A-B had higher frequencies of FoxP3^+^ Tregs and M-Tregs compared with HCs (*p* = 0.0436 and *p* = 0.0092 respectively, Figure 4, A and B). Memory CD8^+^ T cells (M-Tc) were also significantly elevated in A-B compared with those in A-L (*p* = 0.0006, Figure 4C). M-Tc frequencies were associated with A-B (AUC: 0.887; CI: 0.7750–0.9944, *p* < 0.0001) by ROC analysis (Figure 4D).

#### Frequencies of B-Cell Subsets in ALS

**Figure 5:**
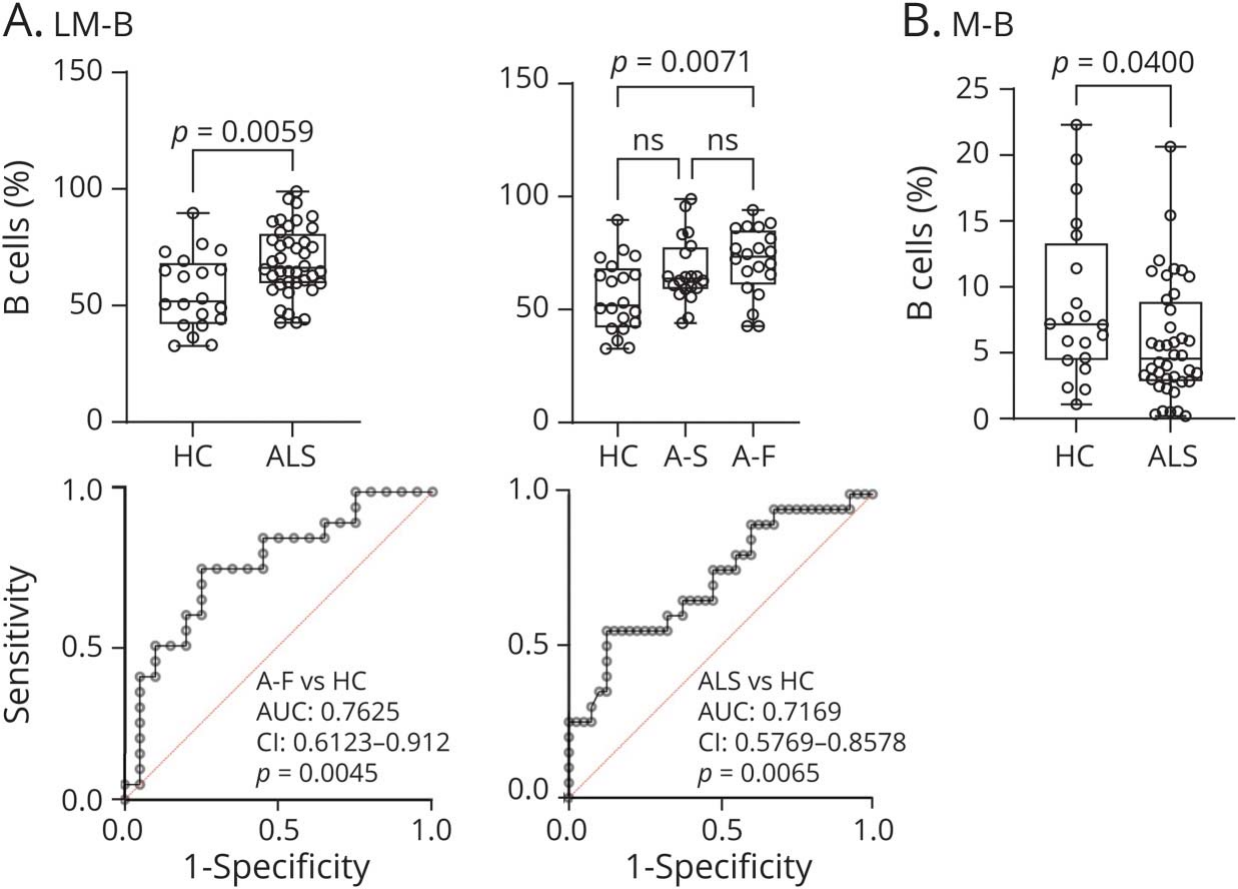
Analysis of B-Cell Subset Frequencies and Their Correlation With Disease Progression in ALS (A) Blood frequencies of LM-B are increased in A-F. The blood expression of LM-B separates ALS from HCs and A-S (ROC analyses). (B) Blood frequencies of M-B are higher in HCs compared with those in ALS. A-F = faster progressing ALS; ALS = amyotrophic lateral sclerosis; A-S = slow progressive ALS; HCs = healthy controls; LM-B = late memory B cells; M-B = memory B cells; N-B = naive B cells; ROC = receiver operating characteristic.

PBMC analysis by FC revealed that LM-B frequencies were significantly elevated in blood from patients with ALS (*p* = 0.0059) and in faster progressing ALS (A-F; *p* = 0.0071) compared with those in HCs (Figure 4A). Analysis of specificity and sensitivity showed that LM-B cells separated patients with ALS from HCs (AUC: 0.7169, CI: 0.5769–0.8578; *p* = 0.0065) and A-F from HCs (AUC: 0.7625, CI: 0.6123–0.9127; *p* = 0.0045; Figure 5A). Memory B cells (M-B) were reduced in blood from patients with ALS compared with those from HCs (*p* = 0.0400; Figure 5B), similarly to switched memory (*p* = 0.0387, data not shown) and follicular B cells (F-B, *p* = 0.0166, data not shown).

### Multiple Linear Regression and Survival Analysis

To estimate the effect of multiple variables on disease progression rate from baseline to the last visit (ALSFRS change), we first fitted a linear model with predefined clinical explanatory variables using multiple linear regression analysis and estimated the effect on survival using Cox regression analysis. The dependent variable for linear regression was the ALSFRS change, while survival from baseline was the outcome of Cox proportional analysis. Clinical variables included age at V1, A-B, ΔFRS, diagnostic delay, ALSFRS-R at V1, and sex (male; Table 2, clinical covariates). Independent variables with a significant effect on ALSFRSR change were ΔFRS and ALSFRS-R at baseline, with estimates (unstandardized coefficients) of 0.661 (CI: 0.531–0.790; *p* < 0.001) and −0.019 (CI: −0.030 to −0.007; *p* = 0.001), respectively. Sex (male) was also associated with ALSFRSR change, but the effect did not reach statistical relevance (*p* = 0.066). These data indicate that higher baseline ΔFRS scores had a positive effect, whereas higher baseline ALSFRS, a negative effect on the disease progression rate (Table 2). In line with previous reports,^32^ Cox regression analysis showed that age at baseline (hazard ratio [HR]: 1.082, CI: 1.032–1.136, *p* = 0.001) and ΔFRS (HR: 3.526, CI: 1.243–10.00, *p* = 0.017) were predictors of shorter survival.

We then added to the model NfL plasma concentrations, M-Tregs, M-Tc, M-B, and LM-B blood frequencies as independent variables (Table 2). Higher frequencies of LM-B were associated with ALSFRS change, although the effect did not reach statistical significance (*p* = 0.078). However, LM-B was a predictor of shorter survival, (HR: 1.094, CI: 1.026–1.167, *p* = 0.006). As previously suggested,^32^ higher NfL plasma concentrations at baseline were associated with ALSFRS change (estimate: 0.661, CI: 0.531–0.790; *p* < 0.014), but unlike previous reports, they were not predictors of survival (Table 2).

We then studied the effect of the same clinical variables on the expression of target lymphocytes. Survival from V1 and ΔFRS had opposite effects on LM-B levels at baseline (estimate: −0.63, CI: −1.046 to −0.22, *p* = 0.003 and estimate: 8.56, CI: 2.64–14.48, *p* = 0.006, respectively), in keeping with the reported association between higher levels of LM-B and a worse prognosis of the disease. ΔFRS had also an effect on M-B baseline frequencies (estimate: 3.86, CI: 1.60–6.12, *p* = 0.001).

We also looked at whether T-cell and B-cell frequencies, dichotomized at the median, were predictive of survival from V1 in a univariate analysis. Above median frequencies of LM-B cells were associated with a significantly reduced survival (30.9 months) compared with below median frequencies (111.3 months; *p* = 0.0306; log-rank χ^2^: 4.675; eFigure 6B, links.lww.com/NXI/A750). Similarly, above median frequencies of M-B and of the cognate-switched M-B were predictors of reduced survival (27.20 months) compared with below median frequencies (51.52 months; *p* = 0.0274; log rank: 4.864, eFigure 6C, links.lww.com/NXI/A750). By contrast, higher levels of F-B were associated with a significantly improved survival (113.3 months) compared with lower levels (38.8 months; *p* = 0.0422, log rank: 4.128; data not shown).

### NfL Analysis

NfL concentration was measured in plasma obtained from blood samples used for CyTOF and FC analysis. A fraction of PBMCs from the same samples has been used for an FC-targeted analysis of monocyte subsets, and the results of NfL analysis have been reported in a published manuscript (doi: 10.3390/ijms23063370). NfL concentration was also tested in plasma from those individuals enrolled in the CyTOF study. Linear regression and pairwise analysis failed to identify any significant association between NfL concentrations and lymphocyte subset frequencies, except for a modest correlation between NfL concentrations and blood frequencies of M-Tc (R: 0.3959; *p* = 0.015) and M-B (R: 0.3928; *p* = 0.0122).

### Longitudinal Analysis

**Figure 6:**
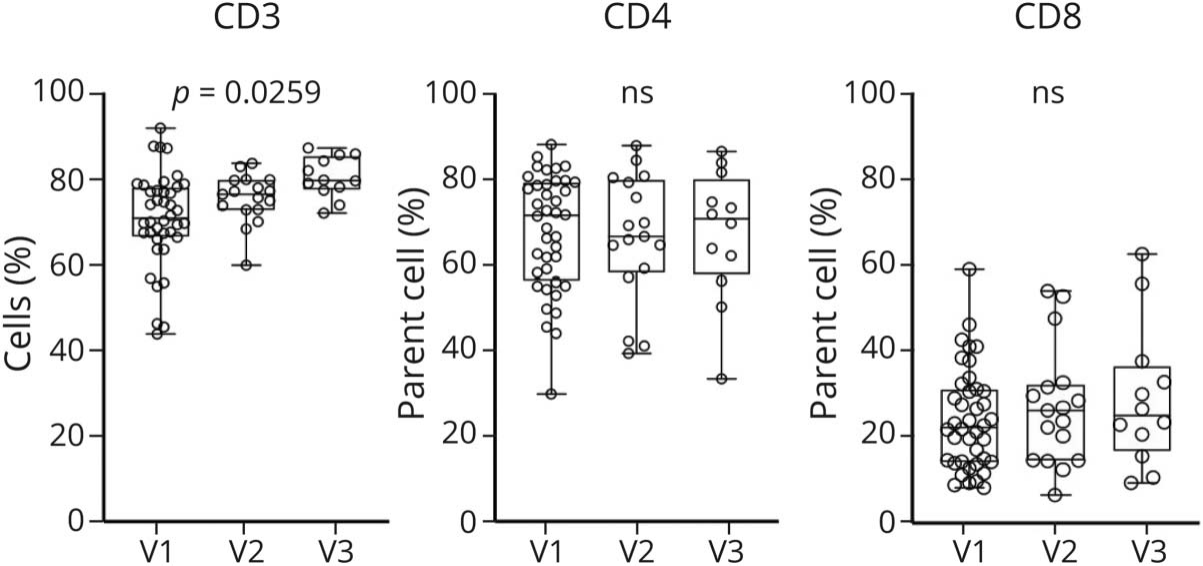
Changes of Lymphocyte Frequencies in Longitudinal Blood Samples Box plot representation of ANOVA for repeated measures analysis (mixed model with missing values at random) of the changes from V1 to V3 showing a significant increase over time of CD3 (*p* = 0.0259) and the relative stability of CD4 and CD8. All remaining T-cell and B-cell subgroups under investigation, including those expressing markers of cell senescence, show variable patterns of expression but no significant changes across time points.

We also investigated changes of blood lymphocyte frequencies across time points using ANOVA for repeated measures (mixed models), comparing baseline frequencies with the later time points. No significant changes in frequencies across time points were detected for B cells and for cells expressing markers of senescence. We identified an increase in the frequency of CD3 T cells from V1 to V3 (ANOVA for repeated measures, *p* = 0.0259), with a significant difference in CD3 frequencies between V3 and V1 (nonparametric ANOVA, Kruskas-Wallis, *p* = 0.0015), while relative proportions of CD4 and CD8 lymphocyte frequencies remained stable across time points (Figure 6).

## Discussion

We report that a significant proportion of T and B cells in blood from patients with ALS display changes indicative of immunosenescence, a mark of immunologic dysfunction increasingly reported in aging, chronic inflammation, and autoimmunity. Using multidimensional CyTOF of blood mononuclear cells and targeted lymphocyte FC analysis, we showed higher frequencies of senescent CD57–expressing CD4 cells in ALS, an association between CD4 CM frequencies and reduced survival and between increased CD95 expressing CD8-cell frequencies and greater neurologic impairment. We also show that increased frequencies of memory Tregs and memory CD8 cells (M-Tc) can distinguish bulbar and fast progressing ALS from HCs. The elevation of baseline LM-B in blood is linked to a reduced survival in a multivariable analysis.

B and T lymphocytes can develop into a state of senescence, which often coexist with downregulated telomerase, shortened telomeres, increased DNA damage response, and increased mitogen-activated protein kinase activity.^36-39^ Viral infections, such as cytomegalovirus, especially in older individuals, can also induce the expression of senescent markers such as CD57 and KLRG1 on T lymphocytes, so does repetitive antigenic stimulation. In our study, the subgroup of patients with ALS with higher CD57 CD4 frequencies (Figure 1C) did not share any specific clinical or demographic characteristic. We could also not confirm that higher frequencies of CD57-expressing senescent T lymphocytes resulted from a persistent exposure to viral infections. Of potential interest, the reactivation in motor cells from patients with ALS and animal models of human endogenous retroviruses (HERV-K) has been recently reported, a biological phenomenon conducive of chronic inflammation in ALS and a promising target of antiretroviral drugs.^40-42^ Confirming previous observations of a distinctive immunologic signature in PBMCs from A-B,^35^ we have identified an upregulation of CD4^+^ FoxP3^+^ Tregs, M-Tregs, and M-Tc cells in blood from patients with ALS compared with that in HCs, with significantly higher frequencies of these lymphocytes in A-B (Figure 3). M-Tc frequencies provide a strong separation of A-B from A-L patients (AUC: 0.8676.; *p* < 0.0001). These markers could be used as means of disease stratification based on disease phenotype in the design of clinical trials. Immunotherapies targeting the reported senescent T cells may be effective in the subgroup of more severely affected bulbar patients.

It has been shown that B-cell depletion does not affect the development of ALS in SOD1 animal models of the disease,^43^ while frequencies of B cells in blood, including M-B cells, do not differ between patients with ALS and age-matched controls.^44^ However, C9orf72 gene loss-of-function in mice models of ALS leads to a severe state of autoimmunity with loss of tolerance for many nervous system autoantigens.^46^ In Alzheimer disease, therapeutic depletion of B cells alone in animal models is sufficient to reduce the burden of A-beta plaques and of inflammatory microglia, reversing behavioral and memory deficits and slowing disease progression.^45^ Our study shows an increase of LM-B cells in patients with ALS compared with HCs (*p* = 0.0059; Figure 4). LM-B cells are termed double-negative B cells (CD19^+^CD27^−^IgD^−^) and described as “atypical.” They have been implicated in age-related chronic inflammation and in autoimmunity.^21^ Loss of the ability to reign in autoimmunity, a characteristic of senescent B (and T) cells, may cause the rise of potentially pathogenic autoantibodies including those to neural antigens. In ALS, we and other have reported the increase of autoantibodies to proteins linked to ALS risk genes, including TAR DNA-binding protein 43, neurofilaments, and dipeptide repeats, the translation product of the mutated C9orf72 gene.^47-50^

It has been shown that T and B cells, especially LM-B, can acquire an SASP, stepping up production and secretion of proinflammatory cytokines such as IL-2, IL-6, IL-8, tumor necrosis factor and interferon-γ.^14^ We could therefore speculate that senescent lymphocyte may contribute to neuronal degeneration and to disease progression in ALS. In our study, we observe that higher frequencies of LM-B cells in blood are associated to a reduced survival, suggesting a potential link between senescent B cells and clinical outcomes (Figure 5). Our longitudinal study in a subset of patients with ALS show a relative stability of senescent T and B cells with disease progression, in contrast with the rise of CD3 levels between baseline and V3 (Figure 6). Senescent immune cells are known to slow their own removal rate, which in turn propagate senescence and create a stable pool of dysfunctional cells in circulation,^e1^ while CD3 increase may be compensatory to the increase of functionally impaired senescent T cells.^e2–e4^

An important limitation of our study is the relatively small longitudinal cohort of patients with ALS, which cannot fully inform on the changing inflammatory environment in ALS and on the real effect that senescence has on the immunopathology of the disease throughout its progression. The effect of senescence and the dynamics of its pathologic effect in neurodegeneration are far from being understood. Further studies are needed to clarify the origin and propagation of this altered immunologic response. Based on our observations and previously reported data, we could speculate that selective lymphocyte depletion targeting, for example, atypical B and M-Tc cells, may be a therapeutic strategy to explore in ALS. Future studies will have to combine a more extended lymphocyte cytometry with the evaluation of the blood microenvironment in ALS and its effect on B-cell and T-cell functionality.

## Glossary

A-B: bulbar ALS
A-F: faster progressing ALS
A-L: limb ALS
ALS: amyotrophic lateral sclerosis
ALSFRS-R: ALS functional rating scale revised
ANOVA: analysis of variance
CD4 T SEN: senescent CD4 cells expressing CD57
CyTOF: cytometry by time-of-flight
F-B: follicular B cells
FC: flow cytometry
FIt-SNE: Fourier transform–accelerated interpolation-based t-Stochastic Neighborhood Embedding
HCs: healthy controls
HR: hazard ratio
IL: interleukin
IQR: interquartile range
KLRG1: Killer Cell Lectin-Like Receptor G1
LM-B: Late memory B cells
M-B: memory B cells
MMI: median marker intensities
M-Tc: memory CD8^+^ T cell
M-Tc: memory CD8^+^ T cells
M-Tc: Tregs and memory CD8 cells
NB GLM: negative binomial generalized linear models
NfL: neurofilament light chain
PBMCs: peripheral blood mononuclear cells
QL: quasi-likelihood
ROC: receiver operating characteristic
SASP: senescence-associated secretory phenotype
SOD1: superoxide dismutase 1
Tregs: T regulatory cells
